# Reference point indentation is not indicative of whole mouse bone measures of stress intensity fracture toughness

**DOI:** 10.1016/j.bone.2014.09.020

**Published:** 2014-12

**Authors:** Alessandra Carriero, Jan L. Bruse, Karla J. Oldknow, José Luis Millán, Colin Farquharson, Sandra J. Shefelbine

**Affiliations:** a Department of Bioengineering, Imperial College London, London SW7 2AZ, UK; b The Roslin Institute and Royal School of Veterinary Studies, The University of Edinburgh, Easter Bush, Midlothian EH25 9RG, UK; c Sanford Children's Health Research Center, Sanford-Burnham Medical Research Institute, La Jolla, CA 92037, USA

**Keywords:** Bone fracture, Bone quality, Bone toughness, Mouse bone, Reference point indentation, BioDent

## Abstract

Bone fragility is a concern for aged and diseased bone. Measuring bone toughness and understanding fracture properties of the bone are critical for predicting fracture risk associated with age and disease and for preclinical testing of therapies. A reference point indentation technique (BioDent) has recently been developed to determine bone's resistance to fracture in a minimally invasive way by measuring the indentation distance increase (IDI) between the first and last indentations over cyclic indentations in the same position. In this study, we investigate the relationship between fracture toughness *K_C_* and reference point indentation parameters (i.e. IDI, total indentation distance (TID) and creep indentation distance (CID)) in bones from 38 mice from six types (C57Bl/6, Balb, *oim*/*oim*, *oim*/*+*, *Phospho1*^−/−^ and *Phospho1* wild type counterpart). These mice bone are models of healthy and diseased bone spanning a range of fracture toughness from very brittle (*oim*/*oim*) to ductile (*Phospho1*^−/−^). Left femora were dissected, notched and tested in 3-point bending until complete failure. Contralateral femora were dissected and indented in 10 sites of their anterior and posterior shaft surface over 10 indentation cycles. IDI, TID and CID were measured. Results from this study suggest that reference point indentation parameters are not indicative of stress intensity fracture toughness in mouse bone. In particular, the IDI values at the anterior mid-diaphysis across mouse types overlapped, making it difficult to discern differences between mouse types, despite having extreme differences in stress intensity based toughness measures. When more locations of indentation were considered, the normalised IDIs could distinguish between mouse types. Future studies should investigate the relationship of the reference point indentation parameters for mouse bone in other material properties of the bone tissue in order to determine their use for measuring bone quality.

## Highlights

• We investigated fracture toughness and reference point indentation parameters from BioDent microindentation in six different mouse bone types.
• Fracture toughness clearly distinguished mouse phenotypes.
• BioDent parameters at mid-diaphysis across mouse types were close to each other making it difficult to discern between groups.
• Increasing the number of indentations along the bone shaft increases the standard deviations within the data reflecting bone's heterogeneity.
• BioDent parameters values are not indicative of stress intensity fracture toughness in mouse bone.

## Introduction

Bone quality is a concern for aged and diseased bone. Ageing and disease degrade the mechanical and structural properties of bone, increasing its vulnerability to fracture and compromising its function. Clinical conditions such as osteoporosis and osteogenesis imperfecta (OI) clearly demonstrate the importance of bone quality on its fracture risk. Many mechanisms can cause poor bone quality: alterations in mineralization [1], mineral crystal size [2], collagen molecular structure [3] and crosslinking [4], fibril orientation [5], [6], non-collagenous proteins [7], bone architecture and geometry [8], and microdamage [9], [10] can all affect bone mechanical properties. Measuring bone toughness and understanding fracture properties of the bone are therefore critical for predicting fracture risk associated with poor bone quality in age and diseases.

Traditionally, bone toughness is expressed in terms of work to fracture, *W_f_*, which measures the bone's capacity to dissipate energy before final failure. However, these measurements are highly dependent on the specimen geometry, the bone matrix structure, and the distribution of defects within the sample. Recent studies, using notched samples described the fracture mechanics of bone in terms of linear elastic stress intensity factor, *K_C_*, a critical value of the toughness characterizing complete fracture of the bone [11], [12]. This fracture mechanics approach significantly improves measurements of toughness as it accounts for geometric characteristics of the sample and reduces the effects of microstructural defects by introducing a notch to represent a worst-case pre-crack.

Despite the recent improvements in measuring bone toughness as a proxy for bone quality, it is still difficult to measure toughness in vivo. To overcome this limitation, Active Life Scientific (Santa Rosa, CA) has developed BioDent, a novel reference probe indentation technique for measuring bone quality. This technique employs a reference probe that rests on the surface of the bone and a conical indentation probe that repetitively indents the bone in a single location. Previous studies have associated the indentation distance increase (IDI) between the first and the last indentations over cyclical indentation in the same position, to the bone's ability to resist additional deformation with repetitive loading, representing therefore a local measure of the post-yield mechanical properties of the bone [13]. Since the reference point indentation was introduced in 2006 [13], several studies on human and animal bone tissue have been carried out to analyse age- [14], treatment- [15], and strain-related [16] differences in values obtained from the BioDent technique. Many of them suggest the IDI value obtained from the reference point indentation to be a useful parameter for assessing bone quality or fracture risk [13], [14], [15], [16], [17]. In a pilot study, Diez-Perez et al. [14] found that the IDI value linearly inversely correlates with crack growth fracture toughness in a total of eight cadaveric samples of five human donors. However, the IDI of a larger cohort of bones of different types needs to be investigated before drawing conclusions on its significance.

Due to its simplicity, minimal invasiveness, and the possibility to conduct tests in vivo in humans or small animals, BioDent reference point indentation is an attractive technique for the evaluation of mouse bone tissue mechanical properties for preclinical testing. However, before this methodology can be successfully applied in vivo, ex vivo tests must be conducted in order to investigate the BioDent reference point indentation reliability and the significance of its parameters in assessing bone quality. Therefore, in this study, we investigate the relationship between fracture toughness *K_C_* and indentation distance IDI, total indentation distance (TID) and creep indentation distance (CID) in mouse bones chosen from several mouse models of healthy and diseased bone, and spanning a range of fracture toughness. Because mouse bone is widely used as a model for human bone diseases and for exploring preclinical treatment strategies, establishing an ex vivo relationship between the BioDent parameters and fracture toughness can offer a novel way of estimating toughness in in vivo mouse bone.

## Material and methods

Bones considered for this study were from four mouse strains and six mouse types (C57Bl/6, Balb, *oim*/*oim*, *oim*/+, *Phospho1* wild type (WT) and *Phospho1*^−/−^), chosen to represent a spectrum of bone toughness, from very brittle (*oim*/*oim*) to ductile (*Phospho1*^−/−^) [1], [18], [19], [20]. Specifically, C57Bl/6 and Balb mouse bones represent low and middle mineralised healthy bone, respectively, and are commonly used as control group (or WT models) [21]. Osteogenesis imperfecta murine (*oim*) is a model of brittle bone disease in humans. Homozygote *oim* (*oim*/*oim*) mice experience spontaneous fracture and many bone deformities, mimicking the moderate–severe condition of OI [3]. Heterozygote *oim* (*oim*/*+*) mice do not have spontaneous fracture and their bone material properties are between WT and brittle *oim*/*oim*, therefore model mild OI [22]. *Phospho1*-R74X-null mutant (*Phospho1*^−/−^) mice are a model of impaired production of phosphate within bone [19]. Ablation of PHOSPHO1 enzyme results in skeletal abnormalities immediately after birth and during juvenile development. Bones are extremely ductile due to poor mineralization [1]. *Phospho1* WT mice are the normal counterpart to the *Phospho1*^−/−^ derived from the C3HeB/FeJ background bred to C57Bl/6 mice [19].

In order to verify our methods, we used femora from three fresh frozen C57Bl/6 mice, all male and 5 months old, whose fracture toughness and IDI values have been previously studied [12], [16]. Femora from seven fresh frozen mice for the remaining types, all male and 7 week old, were thawed and dissected, and used for further analysis.

All mice were maintained in accordance with Home Office (UK) guidelines for the care and use of laboratory animals.

### Fracture toughness

**Fig. 1:**
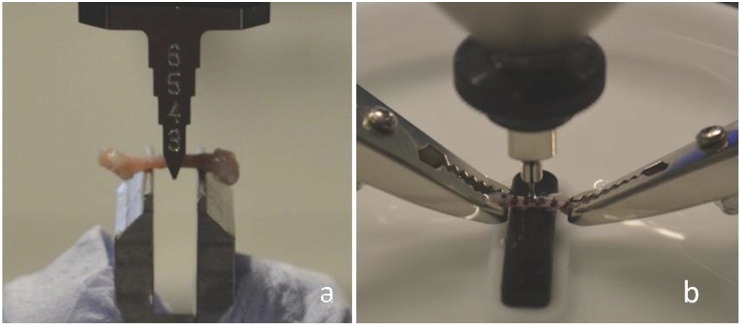
a) 3-point bending fracture test of a notched left femur, constantly kept wet by PBS dropped on its surface, and b) BioDent reference point indentation tests on the mid-diaphysis of the contralateral femur immersed in PBS.

Left femora were notched on the posterior surface at the mid-diaphysis with a razor blade irrigated with 0.5 μm diamond solution [23]. The use of a semi-manual notch-device ensured (i) the depth of the notch to be consistent between the samples and to be ~ 20% of their anterior–posterior width (measured with a calliper), with (ii) an orientation transverse to the mid-shaft such that the nominal crack growth direction was transverse to the long axis of the femur [12], [20]. Each bone notch was then checked under a microscope to ensure notch depth, and notch radius < 10 μm. Bones were stored at 25 °C in gauze wetted with physiological buffer saline (PBS) prior to testing (less than 12 h). In accordance with ASTM standards [23], [24], bones were tested constantly under PBS hydrated conditions in 3-point bending at 1 μm/s displacement rate control (Instron 5800) until complete failure (Fig. 1a). After testing, the fracture surfaces of the bones were examined using an environmental scanning electron microscopy (ESEM) with simultaneous back-scattering (Hitachi S-3400N) in low vacuum mode (pressure of 35 Pa and 25 kV). From these images, geometric characteristics of the bone at the mid-diaphysis were estimated. According to Ritchie et al. [12], the instability fracture toughness, *K_C_*, was estimated using the linear elastic fracture mechanics stress intensity solution for a through-thickness crack in a circular thick-walled cylinder:

(1) $$K_{c}=F_{b}\frac{{PSR}_{o}}{\pi \left(R_{0}^{4}-R_{i}^{4}\right)}\sqrt{\pi \left(\frac{R_{0}+R_{i}}{2}\right)\theta_{\mathrm{inst}}}$$

where *F_b_* is a geometric constant for thick-walled cylinders, *P* is the load at fracture instability, *S* is the span width, *R_i_* and *R_o_* are the inner and outer radii of the bone, respectively, and *θ_inst_* is the instability crack angle [12].

### Reference point indentation parameters: normalised IDI, IDI, TID and CID

Contralateral femora were dissected and stored in PBS wetted gauze at 25 °C before testing (less than 12 h). The bones were indented with the BioDent reference point indentation using a protocol previously used for testing mouse bones [16]. Specifically, femora were submerged in PBS during testing and indentations were applied perpendicular to their anterior and posterior surfaces at five different locations 2 mm distant from each other along the bone diaphysis from the great trochanter to the femoral condyles (Fig. 1b). Indentations were conducted with a maximum indentation force of 2 N, an indentation frequency of 2 Hz, 10 indentation cycles at a touchdown force of 0.1 N [14], [16] using a probe assembly type BP2. Throughout the test the friction of each probe assembly provided was below 0.1 N. Measurements were only considered to be valid if the average maximum indentation force reached the set value of 2 N.

Indentation data was analysed with the BioDent software, which calculated the IDI between the first and the last cycles of indentation as well as TID and CID. Each IDI measurement was then normalised by the IDI value of polymethylmethacrylate (PMMA, Active Life Scientific, Santa Rosa, CA) tested with the same probe (mean of five measurements taken before testing on each bone) in order to eliminate any error introduced by the probe assembly to the IDI measurements [15], [16], [17], [25], [26]. Normalised IDI, IDI, TID and CID were calculated for each sample in multiple locations, i.e. (1) at the anterior mid-diaphysis, (2) as the mean of the indentation in the anterior and posterior mid-diaphysis and (3) as the mean of 10 indentations in five locations of both anterior and posterior sites of the bone diaphysis.

#### Statistical analysis

Normal distribution and homogeneity of variance of the fracture toughness and the BioDent parameters calculated at the bone anterior mid-diaphysis were analysed by the Shapiro–Wilk test and Levene's test, respectively (SPSS, IBM, Somers, NY). Difference in fracture toughness and BioDent parameters (normalised IDI, IDI, TID and CID) at the bone mid-diaphysis between the mouse types were compared using analysis of variance (one-way independent ANOVA for normalised variables and Kruskal–Wallis test for non-normalised variables) and Post Hoc procedures for multiple comparisons. When both anterior and posterior sides of indentation were considered for the normalised IDI measurements, the dependency from the ‘bone side’ was estimated. All tests were two tailed and *p*-values smaller than 0.05 were considered to be significant.

Multilevel linear models were used to statistically compare the normalised IDI values between the mouse types. The data, consisting of 70 measurements per mouse bone type, were organised as hierarchical in four levels with values from the ‘bone location’ (i.e. 1–5 locations) nested within the ‘bone side’ (i.e. anterior and posterior), nested within ‘bone’ (i.e. 1–7 femora per mouse type), and nested within ‘mouse type’ (i.e. 5 mouse strains). In the multilevel linear models, the nesting can inform on any dependency from the nesting (i.e. sides, regions of indentation, specific bone) in determining the IDI value. In particular, the multi-level linear analysis was used in this study as it works with no assumption of homogeneity of the data and because it allows the use of data sets of unequal sizes (i.e. missing data). The latter is important for our modelling as a different amount of IDI indents was considered for each bone, as ideally 10 measurements at 10 sites should be retrieved but some of the indentations had to be discarded when complications occurred, e.g. when the maximum force of 2 N was not reached. The maximum likelihood was assessed to estimate the overall fit of the data to the model, and *p*-values smaller than 0.05 were considered to be significant.

Finally, the R-square correlation coefficient was estimated between BioDent reference point indentation measurements (normalised IDI, IDI, TID and CID for the anterior mid-diaphysis) and fracture toughness *K_C_*. *p*-Values smaller than 0.05 were considered to be significant.

## Results

Normalised IDI values (1.15 ± 0.47 μm) and fracture toughness *K_C_* (4.8 ± 0.18 MPa√m) for the 5 months old C56Bl/6 mouse femora are in well agreement with previous studies [12], [16], indicating that our methods for both techniques were sound.

**Fig. 2:**
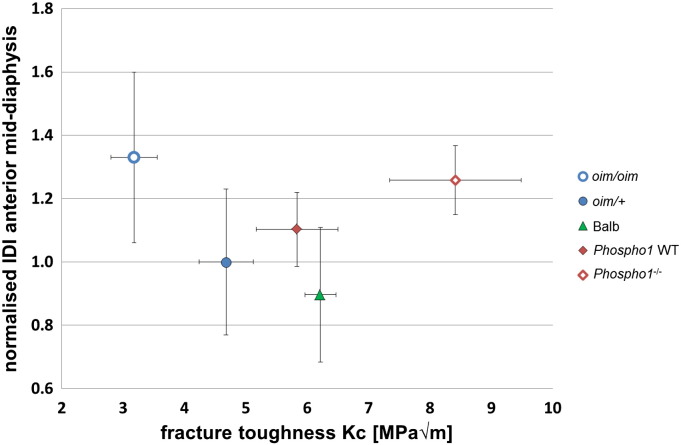
Normalised IDI measured on the anterior mid-diaphysis (mean ± SD) vs. *K_C_* (mean ± SD) fracture toughness values for a group of 35 mouse bones, composed by 5 mouse bone types. The fracture toughness statistically discerned between the different groups (*p* < 0.001), except for the Balb and the *Phospho1* WT group of bones, which are both WT bones. The normalised IDI values were instead overlapping between the different groups, with statistically significant difference found only between *oim*/*oim* and Balb groups (*p* < 0.05).

**Fig. 3:**
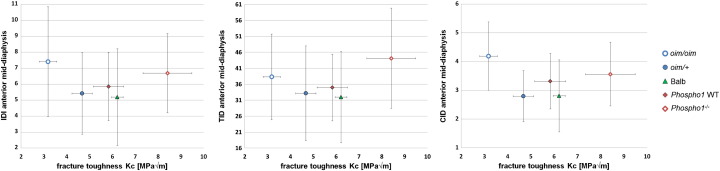
a) IDI (mean ± SD) vs. *K_C_* (mean ± SD) fracture toughness, b) TID (mean ± SD) vs. *K_C_* (mean ± SD), and c) CID (mean ± SD) vs. *K_C_* (mean ± SD), all measured for the anterior mid-diaphysis, for a group of 35 mouse bones, composed by 5 mouse bone types. There was no statistical significant difference between the groups.

Results from notched 3-point bending tests and BioDent reference point indentation for the anterior mid-diaphysis are presented in Fig. 2, Fig. 3.

### Fracture toughness

Results from the fracture toughness tests clearly distinguished between the chosen bone types (*p* < 0.001) except between *Phospho1* WT and Balb, both models of WT bone. Fracture toughness *K_C_* for the *oim*/*oim* bone, model of severe OI, was smaller compared to *oim*/*+*, model of mild OI (mean values are 3.18 and 4.68 MPa√m, respectively). These values were smaller than any other fracture toughness. The *Phospho1*^−/−^, model of hypomineralized flexible bone, had a very high bone fracture toughness, compared to its *Phospho1* WT (the mean values are 8.42 and 5.84 MPa√m, respectively) and any of the other bone type. Balb bones, models of a generic WT bone, had a mid-range *K_C_* value (mean value is 6.22 MPa√m) and overlapped with *Phospho1* WT. Variability (i.e. coefficient of variation equal to standard deviation (SD)/mean) in the *K_C_* data ranged between 4 and 13%.

### Reference point indentation parameters: normalised IDI, IDI, TID and CID

Fig. 2 shows that normalised IDI values for the anterior mid-shaft obtained for the several mouse types were all in the range of 0.90 to 1.33 and showed variability from 9 to 24%, with the Balb and the *oim*/*oim* bones showing the lowest and highest normalised IDI values. Normalised IDI values at the anterior midshaft showed a great overlapping between the groups and differentiated only between *oim*/*oim* and Balb groups (*p* < 0.05, Table 1).

**Table 1:** Normalised IDI values (mean ± SD) of the measurements for the anterior mid-diaphysis, for the average between anterior and posterior mid-diaphysis, and for the average between 10 indentations in five different locations in the anterior and posterior sides along the bone shaft, and fracture toughness *K_C_* for the five mouse bone types, all male and 7 weeks old. Sites of indentation, indicated in red, were 2 mm apart from each other. Fracture toughness was estimated by using 3-point bending techniques, in accordance with ASTM standards [23], [24]. Results show an increase in the SD of the normalised IDI with the number of measurements, reflecting the heterogeneity of the bone. Significance (*p* < 0.05) between groups is indicated by matching letters (e.g. ‘a’ indicates significance between *oim*/*oim* and *oim*/+).

Mouse type	Fracture toughness, *K_C_* (MPa√m) mean ± standard deviation	Normalised IDI mean ± standard deviation		
		Anterior mid-diaphysis	Anterior and posterior mid-diaphysis	10 indentations along the bone shaft
			
*oim*/*oim*	3.18 ± 0.38^a,b,c,d^	1.32 ± 0.24^j^	1.29 ± 0.34^k,l^	1.32 ± 0.42^o,p^
*oim*/*+*	4.68 ± 0.44^a,e,f,g^	1.00 ± 0.23	1.01 ± 0.22^k,m^	0.99 ± 0.31^o,q,r^
Balb	6.22 ± 0.26^b,e,h^	0.90 ± 0.21^j^	0.98 ± 0.24^l,n^	1.00 ± 0.25^p,s,t^
*Phospho1* WT	5.84 ± 0.67^c,f,i^	1.10 ± 0.12	1.10 ± 0.18	1.21 ± 0.30^q,s,u^
*Phospho1*^−/−^	8.42 ± 1.07^d,g,h,i^	1.26 ± 0.11	1.34 ± 0.22^m,n^	1.44 ± 0.45^r,t,u^

When both anterior and posterior sides were considered, the normalised IDI values from *oim*/*oim* and *Phospho1*^−/−^ statistically differed from *oim*/*+* (both *p* < 0.05) and Balb (*p* < 0.005 and *p* < 0.05, respectively). The ‘bone side’ (anterior and posterior) of indentation had no effect on the normalised IDI values.

In Table 1 we report the normalised IDI mean values and SD from (1) the anterior mid-diaphysis, (2) the anterior and posterior mid-diaphysis and (3) from ten indentations in five regions along the shaft in both anterior and posterior sides per each mouse type. Values reported in this table show that increasing the number of indentation locations increased the normalised IDI SD because of the heterogeneous properties of the bone. Indeed, results from the multilevel linear model analysis of the mean normalised IDI values of 10 indentations in five locations along the bone shaft, in both the anterior and posterior sides (~ 57 valid measurements per mouse bone type, for a total of 282 measurements), showed that the side of indentation has no influence but the ‘bone location’ of indentation does have an influence on the normalised IDI values (*p* < 0.05). Statistically significant differences between the normalised IDI values between mouse types were found only when 10 sites per bone were measured (with smaller sampling there was not difference). Table 1 also summaries the statistically significant difference between the mouse types for the normalised IDI values of anterior mid-diaphysis, anterior and posterior mid-diaphysis and of 10 bone locations were considered.

Fig. 3a shows that the IDI values were in the range of 5.19–7.41 μm, TID were in the range of 31.97–44.08, and CID in the range of 2.80–4.19. IDI, TID and CID at mid-diaphysis showed a similar trend observed for the normalised IDI at same location: Balb had the lowest values, and *Phospho1*^−/−^ and *oim*/*oim* the highest ones. IDI, TID and CID values for the anterior mid-shaft obtained for the several mouse types showed a great overlapping between the groups and high standard deviation, and it was not possible to statistically discern between the different bone types.

Correlation between the fracture toughness *K_C_* and the normalised IDI values was very poor at the anterior mid-shaft (R^2^ = 0.000, *p* = 0.920), at the anterior and posterior mid-shaft (R^2^ = 0.000, *p* = 0.917), and at 10 locations along the whole bone (R^2^ = 0.032, *p* = 0.309). Table 2 shows that poor and non-statistical significant correlations were also found between fracture toughness and other BioDent parameters (IDI, TID, CID) measured at the anterior mid-diaphysis for our group of 35 mice represented in Fig. 3.

**Table 2:** Correlation (R^2^ and *p*-value) between fracture toughness *K_C_* and BioDent parameters calculated at the anterior mid-diaphysis for the group of 35 mice (all male, 7 weeks old). The correlation with fracture toughness is also shown with normalised IDI values for anterior and posterior mid-diaphysis and along the shaft of the bone for the same group of mice.

	R^2^ correlation (*p*-value)					
	Anterior mid-diaphysis				Anterior and posterior mid-diaphysis	Whole bone
	Normalised IDI	IDI	TID	CID	Normalised IDI	Normalised IDI
Fracture toughness *K_C_*	0.000 (0.920)	0.003 (0.753)	0.017 (0.459)	0.010 (0.568)	0.000 (0.917)	0.032 (0.309)

## Discussion

BioDent reference point indentation represents an interesting alternative to conventional and destructive mechanical tests for analysing bone quality. In this study we determined if any of the BioDent parameters (normalised IDI, IDI, TID and CID), currently expressing a generic ‘bone quality’ term, correlate with stress intensity fracture toughness, which is another measure of ‘bone quality’ that is mechanically well defined [12]. Results from our cohort of 35 mice with various known fracture toughness showed that values from BioDent reference point indentation very poorly correlate with fracture toughness, *K_C_*, estimated with conventional mechanical experiments. Furthermore, normalised IDI measurements taken at the anterior mid-diaphysis poorly differentiate between groups, but as we increase the indentation locations, the sudden increase in the number of indentations makes the normalised IDI values able to discriminate between the different types of mouse bone investigated.

In this study we estimated fracture toughness using the stress intensity factor formula for a thick-walled pipe geometry section. This formula may introduce several errors due to (i) the approximation of the femoral section of the bone to a thick-walled circular pipe, (ii) the validity of its value at the limit of the small-scale yielding criterion imposed by ASTM standards [23] and (iii) the consideration of its toughness estimation as a plane-stress value [12]. However, because (i) currently no formula exists for calculating *K_C_* as a slightly more elliptical thick-walled pipe, (ii) the ASTM small-scale yielding criterion tends to be quite conservative and (iii) in this study we did not have a widely varying size of bones, the *K_C_* measurements can be considered a valid method for identifying fracture toughness in mouse bone [12]. Indeed *K_C_* was able to differentiate between groups.

Regarding the values for the BioDent parameters obtained from this study, one must consider that these results are highly dependent on the protocol used for the indentation study, i.e. 2 N at 2 Hz for 10 indentation cycles. This was chosen because we had fragile bones (*oim*/*oim*), and we wanted to be able to compare our results with previous studies using BioDent on mouse bones. Our studies conducted on PMMA showed a significant direct linear correlation between average max indentation force and IDI (R^2^ = 0.81, *p* < 0.001) and no significant effect of the number of cycles (10–20 cycles) on the IDI. However, further studies should address the relationship between BioDent parameters and fracture toughness, *K_C_*, in mouse bone using different protocols.

Previous studies using BioDent reference point indentation on cadaveric human bone associated high IDI values to brittle bones and lower IDI to more ductile bones [14]. Specifically, in their preliminary study, Diez-Perez et al. [14] compared the IDI values to crack growth toughness (i.e. slope of the crack-resistance curve, or R-curve) for human samples from five donors (1–3 samples for each donor for a total of 8 samples) and suggested that high IDI value and low crack growth toughness are associated. Another recent study conducted on rat femora and vertebrae, and on dog ribs showed that IDI values correlate with work to fracture, i.e. energy to failure, and that different treatments had significantly different IDI values compared to controls [27]. In our study, we compared the normalised IDI, IDI, TID and CID values to the fracture toughness in terms of stress-intensity *K_C_* at fracture instability, which includes both the contribution of the initiation toughness and the crack growth toughness. The fracture toughness *K_C_* improves measurements of toughness when compared to work to fracture because it considers geometric characteristics of bone and force at ultimate failure, and introduces a notch as a worst-case pre-crack to reduce the effects of microstructural defects within the bone. The samples in our study were chosen to represent a cohort of bone with fracture toughness varying from very brittle to ductile. BioDent parameter values across mouse strains were close to each other, often overlapping, making it difficult to discern differences between mouse groups when a single indentation at the anterior mid-diaphysis was considered. Here, in particular, we show that IDI values for our very brittle bone (*oim*/*oim*) was greater than any others, but also the IDI values for *Phospho1*^−/−^ bone, model of very flexible bone, were higher than the WT and further overlapped the values of brittle *oim*/*oim* bones. Our study is not able to determine if normalised IDI values and other BioDent parameters specifically relate to only the crack growth toughness or to the energy to failure; however it does indicate that normalised IDI, IDI, TID and CID are not indicative of bone fracture toughness *K_C_*. Future studies will need to measure crack growth toughness in mouse bone [20] and investigate its relationship with the BioDent parameters, and to further confirm if any relationship exists between the latter and work to fracture in mouse bone in order to provide a greater understanding of the ‘bone quality’ significance of the reference point indentation parameters.

Although normalised IDI values were not able to predict fracture toughness in our groups of bone, they were able to discern between bone types when multiple locations of indentation were considered. Other material properties of the bone may relate to the normalised IDI value. For example, both stiffness and mineralisation density are known to influence the amount of energy absorbed during failure (fracture energy) [28] and therefore they may have a relationship with the IDI value or other BioDent parameters. This is further supported by the fact that there is no correlation between geometric characteristic of the bone and BioDent parameters (Table 1S, Table 2S in Supplement material), which suggests that material properties, such as fibril and fibre arrangements, lamellar structure, mineralization and porosity architecture are more likely related to IDI, TID and CID. In a very recent work, Ascenzi et al. [29] found a relationship between the BioDent average unloading slope from the first and last loading curves and elastic modulus of collagen density models. Therefore, it is possible that BioDent parameters relate to structural properties of the bone tissue and with strength properties of the bone rather than with toughness. Comparing BioDent parameters and other properties of the bone will be necessary in order to find a definitive significance for the reference point indentation parameters.

In this study, we only compared measurements of BioDent parameters and fracture toughness, *K_C_*, between contralateral femora. This might not be fully representative of other bones of the body but we believe that variability of different bones within the same mouse will be smaller than variability across mouse types examined here.

Bone is naturally heterogeneous and anisotropic because of the great variability in its hydration and in the oriented mixtures of its organic, inorganic and cellular components. In diseases, bone experiences a further increase in heterogeneity of its mineralization and bone matrix organization. Furthermore, the geometry of the mouse bone surface is definitely not perfectly flat. Thus, reference point indentation testing of irregularly mineralised tissue in specimens with imperfectly flat surfaces may therefore result in a high variability of the BioDent measurements for different bone locations. In this study, we compared fracture toughness with the normalised IDI, IDI, TID and CID values for a single measurement taken on each sample at the anterior mid-diaphysis of the bone. However, we also considered the average normalised IDI on the anterior and posterior mid-diaphysis of the same bone and the mean normalised IDI of ten indentations in five locations along the shaft of the femur, in both anterior and posterior side of the bone. We further ran a statistical analysis on the latter normalised IDI values and found significant differences between the mouse types (because of the increased number of measurements), although the SD of the normalised IDI increased with the measurement locations, reflecting the heterogeneity of the bone. Bone ‘location of indentation’ was indeed able to discern between indentations along the bone shaft. Therefore, more consistent results can be obtained by considering a single location of indentation in mouse bone, but these would not be able to discern differences unless many samples are investigated.

The BioDent reference point indentation is different from classical microindentation toughness measurement methods. The latter consider the size of cracks emanating into the bone from a sharp diamond indenter (i.e. a Vickers or cube corner indent) to determine the toughness using an empirical formula, where the toughness is a function of the crack length, elastic modulus and hardness. These methods are however not accurate in quantifying fracture toughness or crack growth toughness or even ranking the relative toughness of most biomaterials [30]. Compared to other materials, bone's composite characteristic can complicate measurements with microindentation; the organic component of bone makes it softer than the brittle materials for which microindentation techniques were intended, and thus prone to even larger errors than normally expected [30]. BioDent reference point indentation does not aim to calculate toughness, but it rather physically measures a value of distance (IDI, TID) as expression of bone quality. Therefore, BioDent reference point indentation differs from conventional microindentation as it does not involve empirical modelling or mechanical assumptions, which often introduce a level of inaccuracy. However, it is necessary to attribute significance to the IDI value in defining the term ‘bone quality’.

## Conclusions

Understanding the quality of the bone is essential for predicting and reducing the fracture risk associated with age and diseases. Novel methodologies for measuring fracture toughness in a less invasive way and in vivo in small animals are of great clinical relevance as they can help to understand material properties of the bone during preclinical testing for reducing fracture risks. However, before these techniques are implemented for in vivo preclinical and clinical testing, future studies should further address the significance of the IDI values or other BioDent indentation parameters for mouse bone in ex vivo models in order to determine its use for measuring bone quality in these small animal bones.

## Conflict of interest

All authors state that they have no conflicts of interest.

The following are the supplementary data related to this article.

Table 1S Geometrical characteristics (mean ± SD) of the femoral mid-diaphysis per mouse bone type as measured after the facture test from ESEM images.

Table 2S Correlation (R^2^ and *p*-value) between geometrical characteristics of the bone very poorly correlated with the IDI and the BioDent reference point indentation parameters at the anterior mid-diaphysis.
