# Supplementary material for: Reference point indentation is not indicative of whole mouse bone measures of stress intensity fracture toughness
Source: Bone. 2014 Dec;69:174–9. doi: 10.1016/j.bone.2014.09.020 (PMC4228060; doi:10.1016/j.bone.2014.09.020)
Supplement: Table 1S — Geometrical characteristics (mean ± SD) of the femoral mid-diaphysis per mouse bone type as measured after the facture test from ESEM images. [file mmc1.doc]

**Supplement material**

| **mouse type** | **outer radius**  **Ro [mm]** | **inner radius**  **Ri [mm]** | **medium radius Rm [mm]** | **wall thickness**  **t [mm]** |
| --- | --- | --- | --- | --- |
| ***oim/oim*** | 0.706 ± 0.067 | 0.544 ± 0.057 | 0.625 ± 0.060 | 0.162 ± 0.028 |
| ***oim/+*** | 0.806 ± 0.060 | 0.575 ± 0.048 | 0.690 ± 0.052 | 0.231 ± 0.033 |
| **Balb** | 0.685 ± 0.032 | 0.456 ± 0.021 | 0.571 ± 0.020 | 0.229 ± 0.036 |
| ***Phospho1* WT** | 0.821 ± 0.049 | 0.599 ± 0.027 | 0.710 ± 0.038 | 0.221 ± 0.025 |
| ***Phospho1*^-/-^** | 0.802 ± 0.073 | 0.565 ± 0.075 | 0.683 ± 0.072 | 0.237 ± 0.035 |

**Table 1S** Geometrical characteristics (mean ± SD) of the femoral mid-diaphysis per mouse bone type as measured after the facture test from ESEM images
