# Supplementary material for: Reference point indentation is not indicative of whole mouse bone measures of stress intensity fracture toughness
Source: Bone. 2014 Dec;69:174–9. doi: 10.1016/j.bone.2014.09.020 (PMC4228060; doi:10.1016/j.bone.2014.09.020)
Supplement: Table 2S — Correlation (R2 and p-value) between geometrical characteristics of the bone very poorly correlated with the IDI and the BioDent reference point indentation parameters at the anterior mid-diaphysis. [file mmc2.doc]

|  | **R^2^ correlation (*p*-value)** | | | |
| --- | --- | --- | --- | --- |
|  | **outer radius**  **Ro** | **inner radius**  **Ri** | **medium radius**  **Rm** | **wall thickness**  **t** |
| **normalised IDI** | 0.002 (0.827) | 0.007 (0.633) | 0.004 (0.732) | 0.006 (0.675) |
| **IDI** | 0.002 (0.830) | 0.003 (0.704) | 0.003 (0.773) | 0.001 (0.896) |
| **TID** | 0.018 (0.454) | 0.014 (0.502) | 0.017 (0.457) | 0.003 (0.748) |
| **CID** | 0.000 (0.958) | 0.002 (0.800) | 0.001 (0.878) | 0.003 (0.752) |

**Table 2S** Correlation (R^2^ and *p*-value) between geometrical characteristics of the bone very poorly correlated with the IDI and the BioDent reference point indentation parameters at the anterior mid-diaphysis.
